# Metastatic renal cell carcinoma patients of T4 stage who are in status of N1 stage or older than 76 years cannot benefit from cytoreductive nephrectomy

**DOI:** 10.1186/s12885-020-07351-w

**Published:** 2020-09-03

**Authors:** Zhao Zhang, Hongliang Wu, Tong Yang, Yaohai Wu, Nengwang Yu, Zhonghua Xu

**Affiliations:** Department of Urology, Qilu Hospital of Shandong University, Wenhuaxi Road 107#, Jinan, 250012 P.R. China

**Keywords:** Renal cell carcinoma, Cytoreductive nephrectomy, Survival, SEER

## Abstract

**Background:**

We aimed to identify which part of the patients with metastatic renal cell carcinoma (mRCC) is not suitable for cytoreductive nephrectomy (CN).

**Methods:**

The data of mRCC patients was acquired from the Surveillance, Epidemiology, and End Results (SEER) database. Multivariate cox regression analysis and nomogram were performed for selecting factors independently associated with survival. Propensity score matching (PSM) was applied to reduce potential bias when comparing survival of mRCC patients treated by CN or non-surgery (NS). The survival analysis of subgroups was estimated by the Kaplan–Meier method and compared by log-rank testing. The summary of subgroup analysis was showed by forest plots.

**Results:**

The records of 21,411 patients with mRCC were obtained from the SEER database. After screening, a total of 6532 patients were included for further analysis, of which 6043 underwent CN and 489 underwent NS. Age, T stage, N stage and tumor size were involved in subgroup analysis by PSM according to the result of multivariate cox regression analysis and clinical experience. Survival benefit was not found in T4 stage patients. Further analysis showed that T4&N1 and T4&age ≥ 76 yr subgroups could not obtain survival benefit from CN.

**Conclusion:**

CN should not be performed in T4 stage mRCC patients who were in status of N1 stage or older than 76 years, because surgery cannot take significant survival benefit for them.

## Background

Renal cell carcinoma (RCC) accounts for approximately 3% of adult malignancies and 90–95% of kidney neoplasms, 25–30% of patients present with metastatic disease at time of diagnosis. Metastatic RCC (mRCC) is one of the most treatment-resistant malignancies and its prognosis is generally poor and median survival after diagnosis is very short [1]. Cytoreductive nephrectomy (CN) was established as a therapy which can improve antitumor immune system response during the era of immunotherapy, given the results of two randomized trials demonstrating an overall survival (OS) advantage of 5.8 months in a combined analysis study [2]. However, targeted therapies (TTs) emerged and demonstrated superiority to immunotherapy, becoming the standard of systemic therapy (ST) in mRCC. The role of CN in mRCC has been questioned because some patients are unable to receive TT after CN due to disease progression or perioperative complications. CARMENA, a randomized controlled trial, suggested treatment with targeted kinase inhibitors (TKIs) alone was not inferior to upfront CN combined with TKIs in certain mRCC patients [3]. For now, it is not well understood if CN should remain a part of the standard treatment protocol.

A research demonstrated that large benefit exists in both overall survival (OS) and progression- free survival (PFS) in patients receiving a CN compared with those without, even after adjusting for imbalances [4]. The latest guidelines suggest that only patients who in the state of International Metastatic RCC Database Consortium (IMDC) favorable and intermediate risk need CN [5]. In order to better understand the role of CN in mRCC, we retrospectively analyzed the records of mRCC in the SEER (Surveillance, Epidemiology and End Results) database and compared the differences in survival among different subgroups treated with CN or non-surgical (NS) after balancing other variables that affected survival.

## Methods

### Data source and patient selection

The SEER database is a cancer-based registry sponsored by the NCI (National Cancer Institute) which incorporates high quality data derived from 18 cancer registries and covers approximately 27.8% of the U.S. population (based on the 2010 census) [6]. We identified patients diagnosed with renal tumor (ICD-0-3 primary site code C64.9) and M1(Derived AJCC M, 6th ed) stage from 2004 to 2015 as mRCC cohort. Patients without exact T stage, N stage, tumor size or survival time were excluded. Patients with code 0 of RX Summ--Surg Prim Site were considered NS group while code 40 and 50 were considered CN group. Only patients who were recommended surgery were involved our research.

### Variables

Variables were obtained using the SEER registries. These variables included sex, race, age at diagnosis, derived AJCC T, 6th ed. (2004–2015), derived AJCC N, 6th ed. (2004–2015), derived AJCC M, 6th ed. (2004–2015), RX Summ–Surg Prim Site (1998+), CS tumor size (2004+), SEER cause-specific death classification, Survival months, vital status recode (study cutoff used), Reason no cancer-directed surgery.

### The process of propensity score matching (PSM)

The main process of PSM was divided into three steps. At first, continuous variables such as age at diagnosis and tumor size were transformed into categorical variables to identify the best cut-off point based on the lowest *P* values and the maximum chi-square of log-rank tests by using X-Tile 3.6.1 software (Yale University) [7]. Secondly, by multivariate cox regression analysis and nomogram plot, variables which can significantly influence cancer specific survival (CSS) of patients were picked out. Thirdly, PSM was performed to reduce potential confounding effects and treatment selection bias by balancing the variables which were selected when comparing CSS between CN and NS in different subgroups. PSM was conducted using the package of “MatchIt” in R version 3.5.3. A 1:2(NS:CN) nearest-neighbor matching with a caliper distance of 0.2 was used.

### Statistical analysis

We compared the cancer specific survival (CSS) among subgroups of mRCC patients treated by CN or NS, using PSM. Then we verified the results by comparing overall survival (OS) between patients treated by CN or NS. Continuous variables were compared with the Student’s t-test. Unordered categorical variables were compared by chi-square tests. Ordered categorical variables were compared by Goodman and Kruskal’s gamma. If the expected frequency was less than five, Fisher’s exact tests were used. The Mann-Whitney U test was applied to compare variables that did have an unnormal distribution. Cumulative survival was estimated by the Kaplan-Meier method and compared by log-rank tests. Statistical analysis was achieved by packages of R software (3.5.3), including MatchIt, tableone and survival. All tests were two sided; *p*-value < 0.05 was considered significant.

## Results

### Data

We identified 21,411 mRCC patients with ICD-0-3 primary site code C64.9 and M1 stage. Among them, 8478 patients were recommended for surgery. Only 7515 patients meted the code of RX Summ--Surg Prim Site 0 (NS), 40 (CN) or 50 (CN). Then 449 pitients with TX and 428 patients with NX were excluded. After that, 106 cases with unknown, abnormal or inaccurate tumor size were excluded (CS tumor size code 0, 600, 700, 800, 900,920,930,950,980,989,990–999). Finally, a total of 6532 cases which met our inclusion criteria were retrieved, including 6043 that underwent CN and 489 cases that received NS. Table 1 showed patient demographics and clinical characteristics of these patients.

**Table 1:** Patient demographics and clinical characteristics for patients of CN and NS group

	CN (***N*** = 6043)	NS (***N*** = 489)	Overall (***N*** = 6532)
**Age (years)**			
Mean (SD)	60.7 (11.5)	69.5 (12.9)	61.3 (11.8)
Median [Min, Max]	61.0 [8.00, 95.0]	71.0 [32.0, 95.0]	61.0 [8.00, 95.0]
**Sex**			
Female	1798 (29.8%)	193 (39.5%)	1991 (30.5%)
Male	4245 (70.2%)	296 (60.5%)	4541 (69.5%)
**Race**			
Black	478 (7.9%)	71 (14.5%)	549 (8.4%)
Other	439 (7.3%)	42 (8.6%)	481 (7.4%)
White	5126 (84.8%)	376 (76.9%)	5502 (84.2%)
**T stage**			
T1	749 (12.4%)	180 (36.8%)	929 (14.2%)
T2	886 (14.7%)	121 (24.7%)	1007 (15.4%)
T3	3926 (65.0%)	134 (27.4%)	4060 (62.2%)
T4	482 (8.0%)	54 (11.0%)	536 (8.2%)
**N stage**			
N0	4243 (70.2%)	324 (66.3%)	4567 (69.9%)
N1	1800 (29.8%)	165 (33.7%)	1965 (30.1%)
**Histologic type**			
Chromophobe	74 (1.2%)	2 (0.4%)	76 (1.2%)
Clear Cell	4582 (75.8%)	401 (82.0%)	4983 (76.3%)
Papillary	314 (5.2%)	14 (2.9%)	328 (5.0%)
other	1073 (17.8%)	72 (14.7%)	1145 (17.5%)
**Survival time (months)**			
Mean (SD)	26.6 (28.6)	11.8 (18.9)	25.5 (28.3)
Median [Min, Max]	17.0 [0, 155]	5.00 [0, 130]	15.0 [0, 155]

### Multivariate cox regression analysis and nomogram plot

**Fig. 1:**
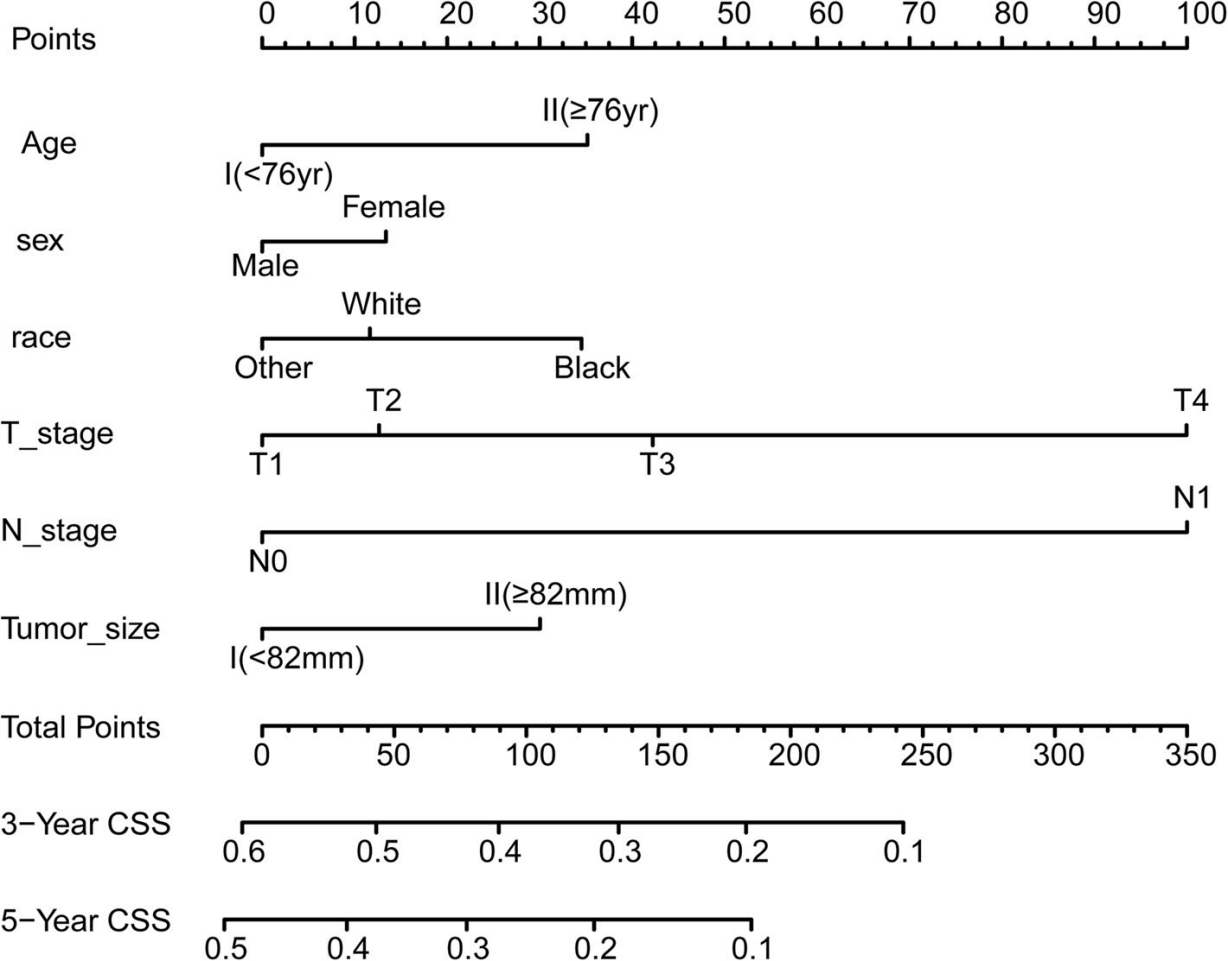
Nomogram plot showed the weight of every variable in the result of multivariate cox analysis

Through X-Tile, we figured out the best cut-off points for age at diagnosis and tumor size as 76-years-old (maximum chi-square 31.3, Miller-Seigmund *P* < 0.0001) and 82 mm (maximum chi-square 45.5, Miller-Seigmund P < 0.0001), respectively. According to the latest guideline and for the convenience of analysis, we unified N2 and N1 in derived AJCC T, 6th ed. (2004–2015) as N1. Nomogram plot in Fig. 1 showed the weight of every variable in the result of multivariate cox analysis. It demonstrated that sex (*P* = 0.01), age (*P* = 0), race (*P* = 0.02), T stage (*P* = 0), N stage (*P* = 0) and tumor size (*p* = 0) were independent risk factors for CSS of total mRCC patients. Therefore, in the next subgroup analysis, the PSM was performed for balancing sex, race, age, tumor size, T stage, and N stage in CN- or NS-treated mRCC patients.

### Subgroup analysis

**Fig. 2:**
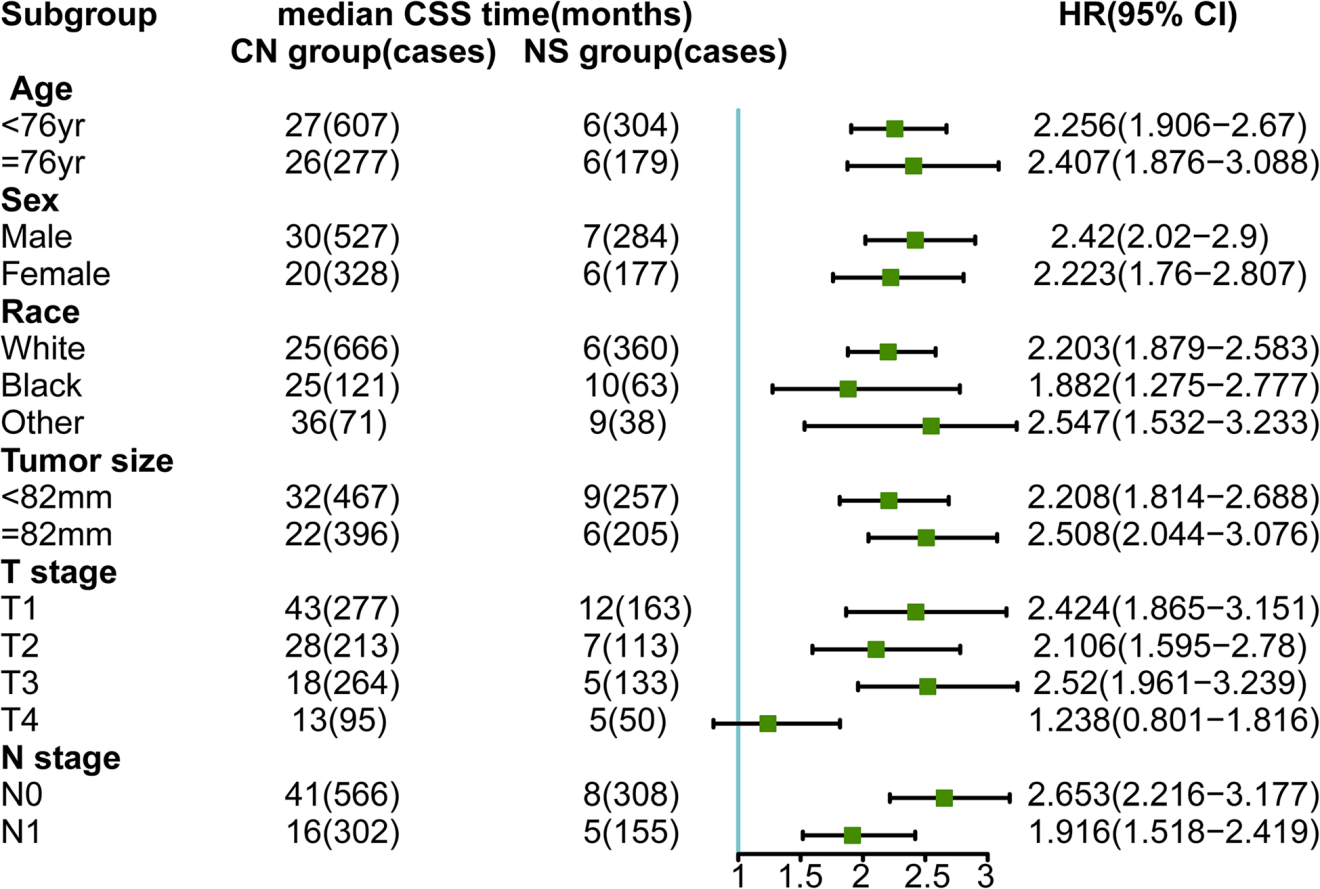
Forest plot of subgroup analysis showed the median CSS time of CN group and NS group and the hazard ratio (HR) between them

First, we compared the CSS of mRCC patients treated by CN or NS for subgrouping under each variable (age, sex, race, T stage, N stage, and tumor size). Other variables were balanced by PSM to reduce potential confounding effects and treatment selection bias when comparing subgroup of one variable. Survival benefits were found in patients treated by CN in almost all subgroups except T4 subgroup. Although there were 8 months benefit of median CSS time in T4 subgroup patients, the HR (95% CI) was 0.801 to 1.816, crossing 1 (Fig. 2). This was consistent with the result of KM survival analysis, showing that in T4 subgroup the CSS between CN (95 cases) and NS (50 cases) had no statistical significance (Fig. 4a, *p* = 0.29).

**Fig. 3:**
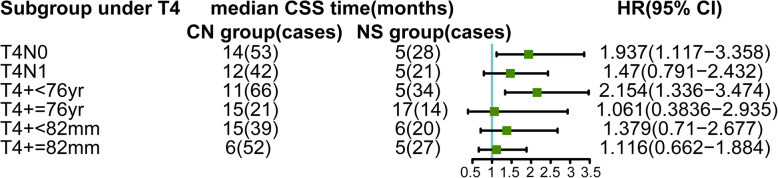
Forest plot of further subgroup analysis under T4 stage

**Fig. 4:**
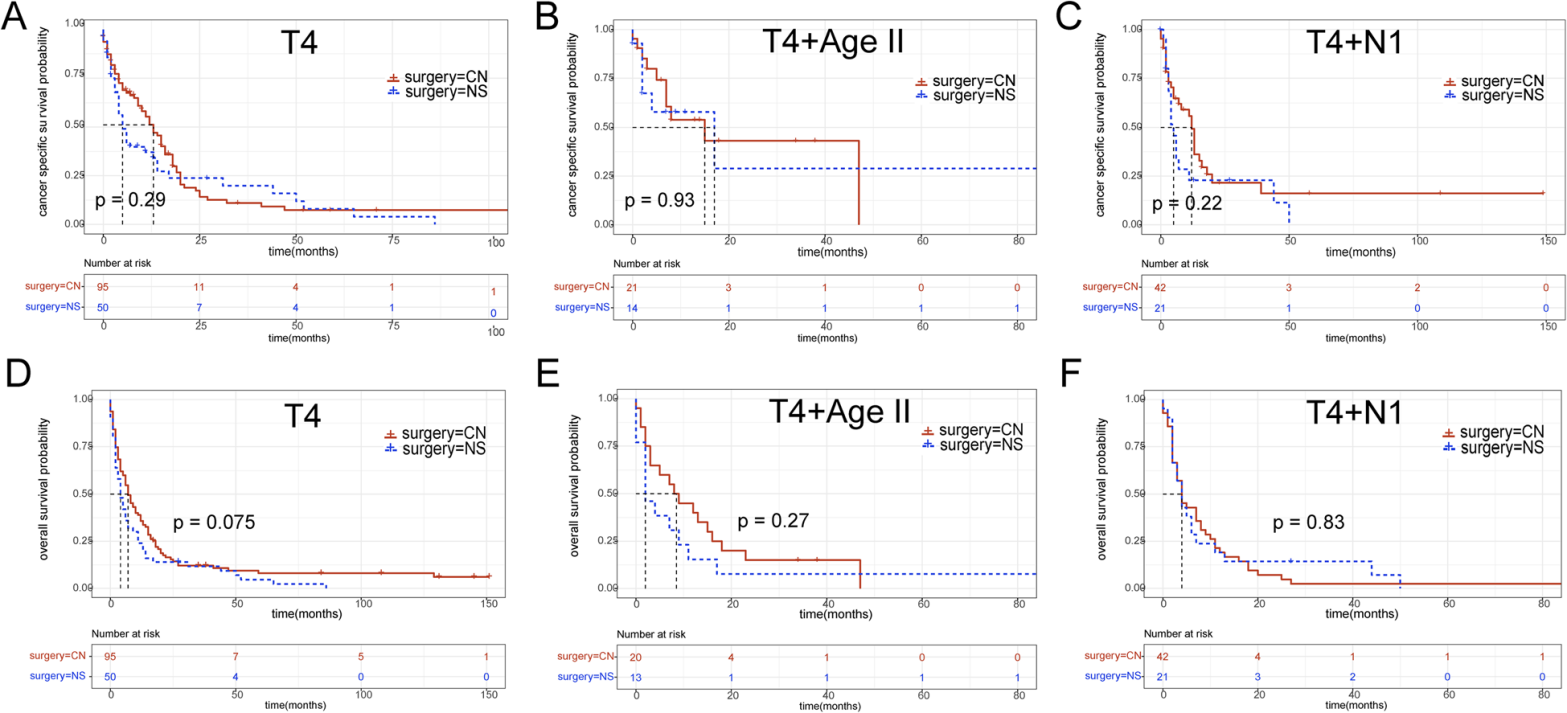
Kaplan-Meier survival curve (OS and CSS) between CN group and NS group in subgroup analysis. (only show T4, T4 + AgeII and T4 + N1 subgroups)

In order to further explore which factors may prevent patients of T4 subgroup from benefiting from CN, we matched T4 stage with age, N stage and tumor size respectively to group them in more detail (Fig. 3). Table 2 showed the result of PSM in T4 stage patients. The result showed that the patients in T4&N0 subgroup and T4&age < 76 yr subgroup could still obtain CSS benefit from CN while T4&N1 subgroup and T4&age ≥ 76 yr could not, meaning N1 and age ≥ 76 yr were high risk factors which influenced prognosis (Fig. 4b, c). In addition, there was no significant difference in CSS between CN- and NS-treated mRCC patients, whether in T4&tumor-size< 82 mm subgroup or T4&tumor-size≥82 mm subgroup. It suggested that although multivariate COX results show that tumor size was an independent prognostic factor, tumor size had little effect on CSS of T4 stage mRCC. At last, we compared OS between patients who received CN or NS in T4, T4&N1 and T4&age ≥ 76 yr subgroups. The result showed the patients of these subgroups also cannot acquire OS benefit from CN (Fig. 4d, e, f).

**Table 2:** The results of propensity score matching (PSM) in subgroups. (only show T4, T4 + AgeII and T4 + N1 subgroups)

	Variables		CN		NS		Significant	
**T4**			No.	%	No	%	P	Method
	N	0	53	55.8	29	58	0.937	chi-square
		1	42	44.2	21	42		
			Mean	SD	Mean	SD	P	Method
	Age (years)		65.2	11.8	65.9	13.6	0.72	t-test
	Tumor Size (mm)		91.2	42.7	88.9	42	0.754	t-test
**T4 + AgeII**			No.	%	No	%	P	Method
	N	0	17	81	10	71.4	0.805	chi-square
		1	4	19	4	28.6		
			Mean	SD	Mean	SD	P	Method
	Tumor Size (mm)		83.8	22.7	82.1	28.8	0.849	t-test
**T4 + N1**			Mean	SD	Mean	SD	P	Method
	Age (years)		62.9	10.9	61.8	13.2	0.721	t-test
	Tumor Size (mm)		86.4	37.5	89.8	39.8	0.742	t-test

## Discussion

Various treatments have been proposed for mRCC. The use of CN in the immunotherapy era was based on two randomized trials conducted by the European Organization for Research and Treatment of Cancer and by the Southwest Oncology Group [8, 9]. In the TT era, CARMENA [3] and SURTIME [10] have greatly weakened the role of CN in the initial treatment of mRCC. CARMENA, as a randomized trial, represents the highest level of evidence to date on the role of CN. However, this trial may be underpowered to detect a true survival benefit because it was enriched with poor-risk patients who cannot obtain a benefit from CN and had a relatively small sample size and an accrual period of 10 years. Besides, it remains unknown whether patients traditionally thought to benefit from CN, such as those with a limited metastatic burden, were actually included in CARMENA. Researches had shown that patients with mRCC displayed significant variability in oncologic outcomes after CN and systemic therapy [11, 12]. Bhindi et al. reported CN may bring survival benefit to patients with limited metastatic burden, well-selected patients, and patients with favorable response after initial systemic therapy or for symptom’s palliation [13]. Larcher et al. found that CN could not offer survival benefit for poor or intermediate risk patients who were eligible for systemic therapy [14]. While these analyses differ in their findings on CN, they do not fully deny CN. They believe that the premise that CN can benefit patients is to strictly limit the indications of CN. Thus, CN likely still has a vital role in the treatment of mRCC patients. It is of great importance to judge which part of patients are not suitable for CN, which can avoid surgery in patients who are unlikely to derive clinical benefit from a surgical intervention.

In the present study, we compared cancer-specific survival among different subgroups of mRCC patients treated with CN or NS, after balancing other variables impacting survival. A special and important inclusion criterion is that all patients must be recommended for surgery by doctors. We found that the CSS of CN patients was better than that of NS patients in almost all subgroups under each variable, which confirmed the value of CN in clinical treatment. Only the patients of T4 subgroup cannot obtain cancer specific survival benefit from CN. Further analysis suggested that age and N stage were two risk factors under T4 stage. T4 stage Patients who were in status of N1 stage or older than 76 years of age cannot benefit from CN. In terms of overall survival, patients in these subgroups were still unable to benefit from CN. Therefore, CN is not suitable for this part of patients. When faced with these patients, we should carefully consider how to choose the most appropriate treatment.

To help oncologists and surgeons assure the best indication for CN and avoid unnecessary surgery, the method of preoperative identification is urgently needed. Previous study has shown that patients with high or intermediate scores can acquire survival benefit from CN under the MSKCC criteria. In addition, according to the ECOG score standard, only patients with a score of 0 or 1 can benefit from CN [15]. Culp et al. previously devised a risk group–based model using seven clinical variables available prior to CN. Patients who had four or more adverse parameters did not appear to benefit from surgery, because their overall survival was similar to the cohort of patients with mRCC who received medical therapy alone [16]. To date, no validated standard can be used to screen mRCC patients who are suitable for CN. To our knowledge, the present study included the largest samples of mRCC patients and focused on subgroup analysis by clinical information for the first time. It is very important that we limit the inclusion to only the patients recommended surgery by doctor can be involved and this This can greatly narrow the selection bias. The results of this study may contribute to future clinical decisions. More randomized controlled trials comparing CN and NS should be conducted and new effective methods for selection of patients should be developed in the future.

The current study exists several limitations. First, SEER data may be limited by unrecorded variables, underreported and incomplete data, variations in data coding and reporting, and migration of patients in and out of the SEER registry area. Second, there was no available detailed information of systemic therapy such as targeted therapy, chemotherapy, and immunotherapy for the cohort of mRCC patients. We did not know whether systemic therapies between the CN and NS groups were balanced. Third, there was no unified criteria for patients who were recommended CN. This may lead to the instability of the included data and affect the final analysis results. Fourth, the selection bias between CN and NS groups could not be completely eliminated by PSM because there was a wide gap in the sample amount between the two groups and the clinical information included in PSM was limited. Fifth, the AJCC TNM stage of our data is 6th edition while several modifications in T and N stages have been made in the 8th edition. When applying the results of this study, the differences between different versions should be taken into account. Sixth, the small sample size of some subgroup after PSM may lead to inaccuracy and randomness of the results.

## Conclusion

CN should be performed in all patients who under T1, T2 or T3 stage because they can obtain survival benefit from surgery. In T4 stage, N1 and age ≥ 76 yr are two high risk factors. Patients who are in T4&N1 subgroup or T4&age ≥ 76 yr subgroup cannot benefit in CSS from CN significantly. In the face of T4 stage mRCC patients, we should choose the treatment therapy carefully and CN is not recommended as the first-line strategy.

## Data Availability

The datasets used and/or analyzed during this current study are available from the corresponding author on reasonable request.

## Abbreviations

mRCC: Metastatic renal cell carcinoma
CN: Cytoreductive nephrectomy
NS: Non-surgery
PSM: Propensity score matching
CSS: Cancer specific survival
SEER: Surveillance, epidemiology and end results database
